# Homework and Academic Achievement in Latin America: A Multilevel Approach

**DOI:** 10.3389/fpsyg.2019.00095

**Published:** 2019-02-01

**Authors:** Rubén Fernández-Alonso, Pamela Woitschach, Marcos Álvarez-Díaz, Andrea M. González-López, Marcelino Cuesta, José Muñiz

**Affiliations:** ^1^Department of Education and Culture, Government of the Principality of Asturias, Oviedo, Spain; ^2^Department of Education Sciences, University of Oviedo, Oviedo, Spain; ^3^Department of Educational and Counselling Psychology, and Special Education, Faculty of Education, University of British Columbia, Vancouver, BC, Canada; ^4^Department of Psychology, University of Oviedo, Oviedo, Spain; ^5^Center for Biomedical Research in Mental Health Network (CIBERSAM), Oviedo, Spain

**Keywords:** homework time, science, academic performance, multilevel models, Latin America

## Abstract

The relationship between homework and academic results has been widely researched. Most of that research has used English-speaking, European or Asian samples, and to date there have been no detailed studies into that relationship in Latin America and the Caribbean. The aim of this study is to examine the effect of quantitative homework characteristics on achievement in science. The sample comprised 61,938 students at 2,955 schools in the 15 Latin American countries (plus the Mexican state of New Leon) which participated in the Third Regional Comparative and Explanatory Study (TERCE), carried out by the Latin American Laboratory for Educational Quality (LLECE) in 2013. The mean age was 12.42 years old (±0.94). Within each country, three hierarchical-linear models were applied at two levels: student and school. The individual level considered time spent doing homework and the school level considered the amount and frequency of homework assignment. In addition, ten control variables were included in order to control the net effect of the characteristics of the homework on the result. The results confirmed that homework is widely assigned in the Latin American region. At the individual level, time spent on homework had little effect on academic performance, while in the quantitative homework characteristics it was the frequency of homework assignment which demonstrated a clearer effect rather than the amount of homework assigned.

## Introduction

Student academic performance is influenced by a broad mix of factors which recent research and reviews have identified: opportunities to learn, time on tasks, classroom organization and management, teaching strategies, learner evaluation and feedback, the school environment, and family involvement and expectations about learning (Scheerens and Bosker, 1997; Scheerens et al., 2007, 2013b; Towsend, 2007; Hattie, 2009; Scheerens, 2016; Fernández-Alonso et al., 2017b). In addition, one must consider the contributions of educational theories originating from sociology which confirm that educational success is largely determined by cultural capital and by belonging to dominant groups (White, 1982; Sirin, 2005; Palardy et al., 2015) as well as the theories of learning which indicate that neurobiological principles, prior knowledge, and cognitive and affective-motivational personality factors are basic components in the formula for academic success (Shell et al., 2010).

Although homework does not feature in the most important factors in the studies cited above, it has attracted a great deal of attention and been the subject of much research as it is the only teaching factor which is done at home. This characteristic of homework fuels social and family debate, and affects other key variables in school performance, such as family involvement, time on tasks, and learning self-regulation.

Research into homework has progressed toward comprehensive models, which include multiple variables related to the characteristics of the homework, teachers, students and their families (Epstein and Pinkow, 1988; Trautwein et al., 2006; Fernández-Alonso et al., 2016). Nonetheless, the aspect which has been studied the most is the relationship between homework time and school results (Goldstein, 1960; Paschal et al., 1984; Cooper, 1989; Cooper and Valentine, 2001; Trautwein and Köller, 2003; Cooper et al., 2006, 2012; Blazer, 2009; Canadian Council on Learning, 2009; Scheerens et al., 2013a; Fan et al., 2017). Despite the mountains of data gathered so far, the results are far from conclusive, as Scheerens et al. (2013a) clearly indicated. They reviewed 128 independent effects of homework time on individual performance with samples in dozens of countries and found varying results: 32% of studies showed negative effects, 33% showed non-significant effects, and 35% showed positive effects. In short, the debate remains open, and there are no simple, unequivocal answers to key questions like whether homework should be assigned or not, or how much time is most appropriate. This apparent contradiction in results, however, is down to two questions that most of the studies we reviewed had not considered. In the first place, when examining the association between achievement and homework time, much of the research had not addressed a key prior question: Why do some students spend longer than others completing their homework? Flunger et al. (2015) identified five student profiles according to time spent and students’ behavior and effort related to homework. In addition, behavior and time spent on homework are conditioned by other variables which also have an influence on school results, such as cognitive capacity, school history, prior knowledge, motivation, sex, age, and sociological factors (De Jong et al., 2000; Trautwein et al., 2002; Trautwein, 2007; Dettmers et al., 2009; Fernández-Alonso et al., 2014, 2015, 2017a). Many studies which have examined the relationship between time spent on homework and school results have not included the effects of these variables in their analyses, hence these apparent contradictions. It is only by controlling for these variables that one may estimate a net effect of the relationship between the quantitative measures of homework and school achievement which is not confounded or affected by other factors.

As indicated by Trautwein and Köller (2003), a significant amount of the research has not addressed the fact that quantitative homework measures are multilevel variables which have different meanings and effects depending on the level being considered. We suppose that the item “How long do you spend on your homework?” when analyzed at the individual level would reflect the student’s dedication and work habits. However, if this item is considered at the classroom or school level, it would tend to be an estimation of the amount of homework assigned. In that case it is capturing the effect of the teachers’ homework policies, a measure with a completely different meaning. In addition, the effect of these two variables on performance is different, the individual measure has little effect on school results (Farrow et al., 1999; De Jong et al., 2000; Dettmers et al., 2010; Murillo and Martínez-Garrido, 2013; Fernández-Alonso et al., 2014; Núñez et al., 2014), and when it is statistically significant, the effect is negative (Trautwein, 2007; Trautwein et al., 2009; Lubbers et al., 2010; Chang et al., 2014; Fernández-Alonso et al., 2015, 2017a; Núñez et al., 2015). This is consistent with the idea that the time spent on homework by the different types of students is not related to school results (Flunger et al., 2015). Multilevel studies, on the other hand, have found positive effects at class and school level when using variables such as frequency and amount of homework (Farrow et al., 1999; De Jong et al., 2000; Dettmers et al., 2009; OECD, 2013; Fernández-Alonso et al., 2017a). It has also been found that when these two variables go together, the frequency of homework has more explanatory power than homework amount (Trautwein et al., 2002, 2009; Trautwein, 2007; Fernández-Alonso et al., 2014, 2015, 2017a). The classical statistical models do not permit the consideration of student and class level effects at the same time. For that reason, it is necessary to use hierarchical-linear models which can separate the effects of the quantitative homework measures into the two levels noted above.

One of the most hotly debated questions is whether the effect of quantitative homework measures is universal or whether there are factors within educational systems which lead to varying effects in different countries, regions and cultures. The amount of homework tends to be higher in Asian countries, whereas the effect of homework on results seems to be more significant in studies in English-speaking and European samples compared to Asian students (Scheerens et al., 2007, 2013a; Dettmers et al., 2009; Fan et al., 2017). There have not been sufficient studies in Latin America to allow conclusions to be drawn in this regard, although it is worth mentioning the work by Murillo and Martínez-Garrido (2013, 2014), the *Third Regional Comparative and Explanatory Study* (UNESCO-OREALC and LLECE, 2016a), and the analysis by Dettmers et al. (2009), which includes the three countries in this region which took part in the second edition of the *Program for Student Assessment* (PISA) in 2003.

Murillo and Martínez-Garrido (2013) used data from nine countries (in addition to Spain), their three level model (student-class-school) did not segregate data by country. The homework variables were reported by teachers and only considered at school level, with neither the amount, nor the frequency being statistically significant. The only positive relationship was between assigning homework and the result in mathematics, but not in Spanish. In their second study Murillo and Martínez-Garrido (2014) once again used measures of frequency and amount of homework reported by teachers, aggregated to school-level. They reported descriptive statistics by country, but the data were not segregated at that level in the hierarchical-linear model, so there is no way to compare effects between countries. Once again they found that neither variable demonstrated a relationship with reading or mathematics results in students in the 3rd–6th years of primary education. UNESCO-OREALC and LLECE (2016a) did compare effects between countries, but the study only looked at one dichotomous variable at the student level reported by parents, not students: spending 30 min or more on homework every day (or not). Finally, the study by Dettmers et al. (2009) is the only one which used quantitative measures at two levels (student and school), although only three countries in the region participated. Once controlled for socio-economic level, the results are rather variable: in Mexico they found positive effects at both levels, in Brazil there was only a positive effect at student-level, and in Uruguay there were no significant effects at either level.

In summary, in the Latin American context, there are no studies which systematically compare the effects of quantitative homework measures using multilevel analysis and control variables. The data available are only general, not segregated by country or strata, and only include quantitative measures at a single level (Murillo and Martínez-Garrido, 2013, 2014; UNESCO-OREALC and LLECE, 2016a), and where those conditions are met, the studies include only a limited number of countries from the region (Dettmers et al., 2009). New intercultural analysis models allow much more rigorous comparisons between countries (Byrne and van de Vijver, 2017). In this context, our current study has two objectives. Firstly, to establish the prevalence of homework in Latin America, describing and comparing the quantitative characteristics of homework in the different Latin American countries. Secondly, to estimate the effects of homework time and characteristics of homework assignment (frequency and amount) on school results, adjusting the analysis models according to the socio-demographics of the students, schools, and countries.

## Materials and Methods

### Participants

The sample population was defined as those students in the 6th grade of compulsory education in 2013 in the 15 participating Latin American countries and the Mexican state of New Leon. In each country the sample was selected following a two-stage stratified cluster method (OECD, 2009; Joncas and Foy, 2012). In the first stage, schools were selected with a probability proportional to their size, and in the second stage a complete class-group was selected from each school, giving a sample of more than 67,000 students. In this current study we excluded students lacking information in the science test, leading to a final sample made up of 61,938 students from 2,955 schools, representing a population of almost 9 million students. The mean age of the students was 12.42 years old with a standard deviation of 0.94. Over two thirds (69.4%) attended state schools, 65.8% attended an urban school; 49.6% were girls, and 81.9% were in the school year corresponding to their age, meaning that the remaining 18.1% had repeated at least one school year at the time of the test.

### Instruments

Two types of instrument were used in the study: (a) tests of academic knowledge, from which we constructed the dependent variable in the study; (b) questionnaires about context for the students, their families, the teachers, and school management, from which we extracted the variables of interest and control variables for our study, with the exception of the relative levels of wealth in each country. The tests were taken during the TERCE evaluation program run by the United Nations Educational, Scientific and Cultural Organization (UNESCO) whose databases are freely available for secondary analysis (UNESCO-OREALC, 2016).

### Tests of Academic Performance

Students completed a battery of tests evaluating reading, mathematics and science, in this study we decided to use the science results as a dependent variable. The science test was produced from a table of specifications organized into five domains and three cognitive processes (UNESCO-OREALC, 2016). It contained 92 items, mostly multiple-choice, grouped in six blocks which were distributed in six test booklets following a matrix design (Fernández-Alonso and Muñiz, 2011). Each student completed one test booklet containing 31 to 33 items they had to answer in 60 min. The items were adjusted to the Rasch model using the Winsteps program (Linacre, 2005). Each student’s score was calculated via the methodology of plausible values, which is the most effective for recovering population parameters in evaluations of education systems (Mislevy et al., 1992; OECD, 2009; von Davier et al., 2009) In TERCE, the individual scores were estimated by combining students’ item responses with information from various co-variables which functioned as imputation factors, they were expressed on a scale with a mean of 700 points and standard deviation 100 (UNESCO-OREALC and LLECE, 2016b).

### Control Variables

When the dependent variable is school performance, it is necessary to include control variables to avoid overestimating the effects of the variables of interest (Fernández-Alonso et al., 2017b). We chose seven control variables from those available in TERCE, all of which are important in the prediction of academic achievement (Liu and Whitford, 2011; UNESCO-OREALC and LLECE, 2016a; Woitschach et al., 2017). Four describe student socio-demographic characteristics: *Gender* (1 = female); *Indigenous* (1 = member of indigenous population); *In paid work* (1 = works and is paid for that work); and *Student’s Socioeconomic and cultural level (SEC)*, a standardized index created by TERCE composed of 17 items about parents’ levels of education, type of work, family income range, amenities and services in the area where they live, and availability of reading material at home. The values of Cronbach’s alpha for this index range between 0.8 and 0.9 depending on the country (UNESCO-OREALC, 2016). The remaining three variables refer to previous school history and the student’s learning resources: *Repetition* (1 = the student has repeated a year during their schooling); *Textbook* (1 = the student has a science textbook); and *Notebook* (1 = the student has a school notebook).

We used three variables to describe the social and demographic context of the schools, two were dichotomous: *School Type* (1 = private school) and whether a school was *Rural*. The third variable was the *School socioeconomic and cultural level*, which was the mean of the student SEC in that school.

### Homework Variables

The questionnaire about student context contained two multiple choice items that were used to construct the variables of interest. Item 1 asked *how many days a week do you study or do homework?* with response options between 0 and 7. Item 2 asked *how long do you spend doing homework on the days when you study?* and had four options: (a) I don’t study; (b) less than 1 h a day; (c) between 1 and 2 h; (d) more than 2 h. The responses were coded as 0, 30, 90, and 150 min, respectively.

These items were used to construct four variables: *Does no homework* (NoHW), a dichotomous variable where 1 indicates students who do not do homework; *Homework time* (HWTime), the mean daily minutes spent doing homework calculated as follows: *HWTime = Item1^∗^Item2/7*. HWTime was squared in order to add a quadratic element to the regression (*HWTime_2*). Teachers’ homework policies were described with two variables: The amount of homework (*HWAmount*), the mean homework time per school; and Frequency of homework assignment (*HWFreq*), the mean number of days in a school that students do homework.

### Procedure

The cognitive tests were applied by expert personnel who were not employed by the school being tested. Tests were carried out on 2 days, the first day for reading and writing, and the second for mathematics and science. The tests for each subject took between 45 and 60 min, with a 30-min break in the middle; following that, after a 15-min break, the student context questionnaires took about 45 min to complete. The questionnaires for the schools, teachers and families were distributed on the first day, and collected at the end of the second day. UNESCO ethical guidelines were followed, and the families of the students selected to participate in the evaluation were informed about the study by the school administrations, and were able to choose whether those students would participate in the study or not.

### Data Analysis

The first step in the analysis was to calculate the descriptive statistics for all variables. Following that, for each country three random-intercept hierarchical-linear models were created with two levels: student and school. The modeling strategy was as follows: first produce a null model without predictors to check the distribution of variance in each level. The second model included the four homework variables, and the third model added the control variables described previously. We used the maximum likelihood estimation method with robust standard errors using the HLM 7.01 program (Raudenbush et al., 2011). In all analyses we used the weightings provided by TERCE which were designed so that each country, regardless of size, would have an equal contribution in the analysis of results (UNESCO-OREALC, 2016), with the sum total of weights in each country being equivalent to 5000 students.

The amount of missing data in the variables ranged from 2 to 12%. We used a two-step strategy to recover missing data. Firstly, the incomplete cases were imputed with the mean of the subject, then the completely missing data were recovered using the iterative EM method with auxiliary variables in the Missing Value Analysis module of SPSS 24. Fernández-Alonso et al. (2012) found that this two-step strategy produces the best recovery of population data in studies with this (non-random) type of missing data and levels of missing data similar to those in TERCE.

## Results

Table 1 shows the data related to the first research objective, the two basic characteristics of homework assignment habits in each country.

**Table 1:** Frequency and amount of homework in Latin American countries.

Country	Frequency: days with homework (median)	Amount: total daily minutes		Percentage not doing homework
		Mean	*SEM*	
Argentina	4	46	0.6	10%
Brazil	4	45	0.5	8%
Chile	3	36	0.5	9%
Colombia	5	57	0.5	2%
Costa Rica	3	42	0.5	5%
Ecuador	5	68	0.6	2%
Guatemala	5	61	0.6	2%
Honduras	5	60	0.6	4%
Mexico	5	50	0.5	4%
Nicaragua	5	60	0.5	4%
Panama	5	60	0.6	5%
Paraguay	4	48	0.5	6%
Peru	5	65	0.6	2%
Dominican Rep.	5	72	0.6	4%
Uruguay	4	45	0.5	11%
New Leon	5	52	0.5	3%

The median number of days doing homework (between 4 and 5 in almost all countries) indicates that Latin American teachers set homework most days of the week. The median is less than 4 in only two cases (Costa Rica and Chile). For the combined TERCE data the estimated amount of homework is a little more than 50 min a day, the equivalent of a weekly volume of a little more than 6 h. However, there are huge variations between countries. For example, homework in the Dominican Republic requires 3.5 h a week more than in Chile. The final column shows the percentage of students who do not do homework. The correlation between this percentage and the amount of homework by country is negative (*r*_xy_ = −0.66), in other words countries with a smaller amount of homework tend to have a higher proportion of students who report not doing homework.

In the hierarchical-linear models, the effect of homework is small in Latin American countries (Table 2). In most countries not doing homework has a negative effect, which is statistically significant (*p* < 0.10) in half of the cases. In general the effect of homework time is not significant when considered at the individual level. Of the five statistically significant cases, four were positive and the other negative. Nevertheless, homework time has a small effect. In Ecuador, for example, where the positive effect is largest, once the control variables are added, the model predicts a gain of less than 8 points for each extra hour spent on homework. In the variables which describe the teachers’ homework policies, the effect of the frequency of setting homework is positive in most countries and statistically significant in six cases. The amount of homework set exhibits mainly small, negative effects that are not statistically significant. The introduction of control variables in model 3 does not change the direction of the effects but it does mitigate them somewhat, with some cases losing statistical significance.

**Table 2:** Regression coefficients and SE of homework variables in models 2 and 3.

	Model 2: HW variables only								Model 3: with control variables							
	Level 1 variables (individual)				Level 2 variables (school)				Level 1 variables (individual)				Level 2 variables (school)			
	No HW		HW Time		HW Freq		HW Amount		No HW		HW Time		HW Freq		HW Amount	
	β	*SE*	β	*SE*	β	*SE*	β	*SE*	β	*SE*	β	*SE*	β	*SE*	β	*SE*
Argentina	**−13.80**	**8.27**	**−**0.09	0.07	**32.73**	**11.15**	**−**0.39	0.55	**−**12.70	7.95	**−**0.08	0.07	3.51	8.10	0.18	0.46
Brazil	9.45	12.19	0.09	0.08	**52.50**	**12.80**	**−1.77**	**0.78**	8.26	11.43	0.05	0.07	**23.87**	**11.08**	**−**0.67	0.71
Chile	**−**7.31	7.82	0.05	0.09	**43.58**	**14.80**	**−**0.64	0.96	**−**4.26	7.61	0.05	0.09	**49.00**	**12.90**	**−1.95**	**0.85**
Colombia	8.33	19.26	0.11	0.08	**62.05**	**20.99**	**−**0.33	0.72	5.59	18.68	0.09	0.09	28.30	20.16	**−**0.80	0.68
Costa Rica	**−**9.75	9.36	**−0.19**	**0.07**	3.01	10.64	**−**0.27	0.59	**−**12.01	8.64	**−0.17**	**0.07**	4.22	7.99	**−**0.39	0.46
Ecuador	**−22.30**	**11.55**	**0.15**	**0.04**	**−**6.03	18.33	0.40	0.39	**]-**14.70	11.42	**0.13**	**0.04**	**−**0.70	14.30	**−**0.06	0.38
Guatemala	**−23.62**	**11.46**	0.01	0.04	**37.79**	**9.45**	**−**0.63	0.41	**−19.27**	**10.67**	0.00	0.04	**20.73**	**8.75**	**−0.65**	**0.39**
Honduras	**−28.39**	**11.32**	0.09	0.06	18.27	11.58	**−**0.13	0.51	**−23.73**	**11.70**	0.06	0.06	**21.29**	**11.21**	**−**0.50	0.52
Mexico	**−17.45**	**9.13**	0.10	0.07	**23.68**	**11.62**	**−**0.59	0.53	**−**12.80	9.29	**0.10**	**0.06**	**15.99**	**8.50**	**−**0.33	0.43
Nicaragua	3.28	9.73	0.02	0.05	13.75	11.99	**−**0.46	0.57	1.85	9.74	0.00	0.06	19.30	13.55	**−**0.73	0.55
Panama	**−**11.25	12.48	0.05	0.07	**−**4.49	12.23	**1.00**	**0.59**	**−**5.60	12.17	0.05	0.07	7.22	10.63	**−**0.04	0.52
Paraguay	**−**12.55	12.52	0.02	0.07	6.34	11.26	**−**0.51	0.57	**−**14.30	12.63	0.00	0.07	**−**0.04	11.54	**−**0.26	0.58
Peru	**−34.82**	**17.61**	**0.11**	**0.05**	7.87	11.38	0.69	0.47	**−32.65**	**17.16**	**0.11**	**0.05**	10.61	8.73	0.42	0.36
Dominican Rep.	**−37.87**	**12.38**	**0.07**	**0.04**	13.68	12.83	**−**0.20	0.50	**−34.19**	**12.14**	0.02	0.04	0.68	9.24	0.07	0.37
Uruguay	**−28.96**	**15.50**	0.05	0.17	**−**22.22	16.75	**1.97**	**1.08**	**−**20.26	13.34	0.05	0.15	**−22.85**	**9.55**	**1.21**	**0.60**
New Leon	**−**12.72	8.78	**0.12**	**0.06**	7.64	10.81	**0.98**	**0.59**	**−**12.38	8.64	**0.12**	**0.06**	**17.32**	**8.61**	0.24	0.46

*SE, standard error; bold text indicates statistically significant effects with at least p < 0.10.*

Table 3 shows the effects of the control variables in model 3. The most determinative variable is socioeconomic level, which is significant in all countries at the individual level, and in almost all countries at school level. Individual variables which stand out include repeating school years which has a negative effect in all cases, and availability of basic learning resources (science textbook and school notebook). Once the effect of those variables is controlled for, the variables of gender, being indigenous, and being in work have a smaller effect. A similar situation occurs with the type of school and whether it is urban or rural, which do not demonstrate statistically significant effects in most cases, probably because their effects are overshadowed by the dominance of the effects of the schools’ socioeconomic and cultural levels.

**Table 3:** Regression coefficients and SE for the control variables.

	Level 1 variables (individual)														Level 2 variables (school)					
	SEC		Female		Indigenous		Repeat year		Working		Science textbook		Notebook		Type		Rural		Mean SEC	
	β	*SE*	β	*SE*	β	*SE*	β	*SE*	β	*SE*	β	*SE*	β	*SE*	β	*SE*	β	*SE*	β	*SE*
Argentina	**10.24**	**3.02**	0.15	3.87	−9.57	7.04	−**31.54**	**4.09**	−16.96	12.64	4.75	4.68	**24.27**	**4.71**	**20.60**	**10.06**	**25.37**	**9.47**	**57.06**	**10.26**
Brazil	**25.79**	**3.48**	−6.09	5.52	2.77	10.09	−**24.97**	**4.99**	−9.05	9.39	**19.56**	**4.62**	**9.71**	**5.32**	12.97	9.93	12.43	11.45	**66.11**	**10.92**
Chile	**13.99**	**3.44**	−0.21	3.73	2.54	5.76	−**29.99**	**5.83**	2.90	12.28	**35.20**	**5.11**	−0.07	4.66	9.18	9.92	9.12	15.84	**66.49**	**7.30**
Colombia	**13.86**	**4.41**	−**10.21**	**5.49**	8.05	11.99	−9.31	6.69	−27.98	24.31	−5.61	6.44	**14.31**	**4.71**	−2.67	10.01	12.66	13.10	**50.05**	**10.22**
Costa Rica	**17.19**	**3.27**	−**11.41**	**3.72**	−7.47	9.56	−**29.22**	**3.63**	6.54	13.91	3.02	4.93	**11.03**	**5.30**	**36.88**	**13.59**	6.14	25.19	**38.48**	**9.41**
Ecuador	**17.64**	**2.46**	−5.44	3.68	2.75	6.84	−**16.91**	**4.23**	−1.61	8.18	**16.62**	**4.98**	6.18	4.96	−16.75	13.25	25.69	18.45	**67.37**	**9.64**
Guatemala	**12.08**	**2.30**	−**10.79**	**4.15**	**8.99**	**5.08**	−**15.75**	**3.62**	−9.15	6.03	−6.85	5.66	**10.34**	**4.40**	**38.37**	**12.47**	2.62	8.16	**48.77**	**8.13**
Honduras	**13.66**	**3.48**	−0.21	3.70	1.36	7.05	−**17.48**	**3.67**	−5.89	10.82	−**12.87**	**5.78**	**13.87**	**4.68**	26.75	21.98	−14.79	17.88	19.10	14.46
Mexico	**17.39**	**3.09**	−1.15	3.35	1.76	6.93	−**16.69**	**5.66**	−5.85	8.38	**24.55**	**4.57**	8.16	5.36	11.31	13.34	14.25	11.08	**47.93**	**8.24**
Nicaragua	**9.53**	**1.71**	−**7.88**	**2.47**	−0.02	7.80	−**9.31**	**3.73**	0.66	7.96	−**7.48**	**3.48**	**8.46**	**3.74**	21.58	13.28	5.57	15.91	**32.05**	**14.09**
Panama	**15.75**	**2.76**	4.49	3.88	2.84	5.04	−**8.36**	**4.51**	−9.36	11.77	**14.67**	**4.21**	9.38	6.27	7.97	11.04	8.61	10.73	**55.92**	**8.20**
Paraguay	**8.22**	**2.61**	3.85	3.62	−7.61	6.96	−**8.39**	**3.71**	−3.26	8.44	1.00	4.56	**17.72**	**6.74**	**38.81**	**13.60**	−11.58	15.21	**27.29**	**11.97**
Peru	**11.21**	**2.29**	−5.53	3.45	−5.40	5.13	−**19.14**	**3.47**	−6.45	6.63	5.99	4.37	**10.82**	**4.40**	**38.24**	**10.16**	−**49.98**	**11.50**	11.97	8.80
Dominican Rep.	**21.42**	**2.79**	−0.72	3.93	NA	NA	−**15.40**	**3.07**	−1.71	7.28	**19.22**	**4.26**	4.90	4.24	9.81	6.33	8.62	7.32	**45.80**	**6.51**
Uruguay	**28.70**	**4.86**	−11.72	7.98	18.24	14.84	−**42.97**	**8.21**	−39.19	24.84	10.74	7.97	17.36	12.0	**39.57**	**20.50**	**86.87**	**21.22**	**58.13**	**19.03**
New Leon	**5.23**	**2.20**	0.83	3.54	−6.01	7.94	−**12.29**	**4.27**	−7.07	6.29	**7.54**	**4.41**	2.86	4.88	**20.72**	**11.09**	−**16.54**	**9.20**	**29.21**	**6.80**

*SE, standard error; NA, not apply. In Dominican Republic “Indigenous” was not considered. Bold text indicates statistically significant effects with at least p < 0.10.*

Table 4 shows the distribution of the percentage of variance between the two levels of analysis in model 1 (without predictors) and the percentage of that variance explained by models 2 and 3. The percentage of variance in model 1 at school level (L2) indicates that there are significant differences between schools in Latin American countries. One group of countries (Ecuador, Guatemala, Honduras, Nicaragua, Paraguay and Peru) have approximately 50% of the variance in level 2, whereas in those cases where the variance is smaller, it is around 20% (Costa Rica and the Dominican Republic). The results indicate that the percentage of total variance explained by the homework model (model 2) is small, when it is not practically null. In countries where the effect of the homework variables is greater (Argentina and Colombia), the reduction of the total variance is about 7%, but in other cases (Costa Rica, Ecuador, Nicaragua, and Paraguay) the data explain less than 1% of the total variance. Furthermore, the reduction of variance between students is very small in all cases, which confirms that quantitative homework variables have more impact on the differences between schools than the differences between students. Finally, model 3 indicates that the control variables explain more than half of the variance between schools in most cases and between 15 and 30% of the total variance.

**Table 4:** Distribution of the variance in model 1 and percentage of variance explained in models 2 and 3.

	Model 1: without predictors					Model 2: homework variables			Model 3: all variables		
	L1	L2	Total	% Var L1	% Var L2	L1	L2	Total	L1	L2	Total
Argentina	5663.5	3241.0	8904.5	64%	36%	0.3%	18.0%	6.8%	5.8%	64.4%	27.1%
Brazil	6174.2	2878.5	9052.7	68%	32%	0.2%	15.3%	5.0%	8.3%	72.2%	28.6%
Chile	9021.2	3745.2	12766.4	71%	29%	0.2%	15.4%	4.6%	4.6%	67.0%	22.9%
Colombia	6430.3	2637.1	9067.4	71%	29%	0.1%	23.8%	7.0%	2.8%	52.5%	17.3%
Costa Rica	5786.7	1756.0	7542.7	77%	23%	0.3%	−0.8%	0.0%	6.6%	57.7%	18.5%
Ecuador	4518.4	3962.2	8480.6	53%	47%	0.6%	0.7%	0.6%	3.9%	42.8%	22.1%
Guatemala	3275.0	3213.8	6488.8	50%	50%	0.1%	8.8%	4.4%	4.1%	72.9%	38.2%
Honduras	3306.9	3119.8	6426.7	51%	49%	0.8%	7.2%	3.9%	3.7%	30.2%	16.5%
Mexico	5199.3	2484.6	7683.9	68%	32%	0.3%	5.6%	2.0%	6.1%	61.0%	23.8%
Nicaragua	2586.3	2547.0	5133.3	50%	50%	0.1%	1.2%	0.6%	1.6%	26.6%	14.0%
Panama	5333.2	3005.1	8338.3	64%	36%	0.0%	5.6%	2.0%	3.7%	65.3%	25.9%
Paraguay	3824.2	4062.0	7886.2	48%	52%	0.0%	0.1%	0.1%	1.8%	26.1%	14.3%
Peru	4041.0	4049.1	8090.1	50%	50%	0.6%	12.2%	6.4%	4.1%	54.0%	29.1%
Dominican Rep.	4154.4	1035.3	5189.7	80%	20%	1.3%	10.7%	3.2%	9.5%	65.1%	20.6%
Uruguay	8959.3	3117.9	12077.2	74%	26%	1.3%	7.0%	2.8%	11.6%	81.1%	29.6%
New Leon	5638.0	2188.9	7826.9	72%	28%	0.2%	11.8%	3.5%	1.2%	48.4%	14.4%

*L1, Level 1 variables (individual); L2, level 2 variables (school).*

## Discussion and Conclusion

There are three main reasons justifying interest in this work. The first is its general scientific character: the effect of quantitative homework variables on school results is something which has been widely researched (v. g., Goldstein, 1960; Paschal et al., 1984; Trautwein and Köller, 2003; Cooper et al., 2006, 2012; Trautwein et al., 2006; Scheerens et al., 2013a; Fan et al., 2017) but which has not produced a unanimous answer which is why it is important to add new evidence in that regard. Nonetheless, in the context of Latin America and the Caribbean, a region which represents approximately 8% of the world’s population, there are no studies which focus on systematically analyzing this topic. Research available up to now has not presented data separated by country and has only assessed homework variables in aggregate (Murillo and Martínez-Garrido, 2013, 2014; UNESCO-OREALC and LLECE, 2016a). The second justification is the need to examine whether the results from the research cited above are also found in the Latin American context, which will let us see for the first time the prevalence of homework in those countries, and look at the possible differences between countries. In other words, allow us to analyze the invariance of the relationships in the various Latin American countries (Byrne and van de Vijver, 2017). This study aims to provide transcultural validity by offering data which can be compared with the evidence accumulated by studies in English-speaking, European and Asian populations. Finally, the third reason is that our research may serve as a guide and a stimulus for other similar research in Latin American countries.

If we consider the first objective, we can conclude that more than 90% of Latin American and Caribbean students do homework to some extent, which is comparable with Western and Asian countries (Dettmers et al., 2009; Fan et al., 2017), and which seems to confirm that homework assignment is a universal teaching resource. The amount of daily homework in each region is highly variable. For example, in the Dominican Republic students report spending twice as long on homework as in Chile. Nonetheless, the time spent on homework in most countries ranges between 45 and 60 min a day, which is in line with what one would expect for students in the 6th grade according to Cooper (2001) “10 min rule.”

Previous evidence from multilevel analyses indicated that the effect of homework time at the individual level is small and when it is statistically significant, this effect is negative (Trautwein, 2007; Dettmers et al., 2010; Núñez et al., 2014, 2015). These results seem to be confirmed in Latin America and the Caribbean, as in the model with control variables only Ecuador, Mexico (including New Leon) and Peru gave results contrary to that hypothesis. In the case with the greatest effect (Ecuador), the model predicts gains of less than 8% of a standard deviation for each extra hour spent on homework; little yield for the effort and dedication needed.

In general, the quantitative variables describing teachers’ homework policies produce expected results, although the proportion of statistically significant effects is rather lower than one might expect based on the evidence available from other contexts. After applying the control variables, only half of the countries demonstrated statistical significance for the frequency or amount of homework set. Nevertheless, these data are consistent with previous research indicating that the frequency of homework seems to have more impact on results than the amount of homework set (Trautwein et al., 2002, 2009; Trautwein, 2007; Fernández-Alonso et al., 2014). These results have clear educational implications for teachers’ homework policies, as they seem to indicate that the frequent assignment of homework has more positive effects than assigning large amounts of homework.

It is worth noting that the effects of homework frequency and homework amount on scores in science are closely related as the correlation between these effects is very negative (*r* = −0.88). This would seem to indicate that in those countries where frequency has less influence, homework amount has a greater effect. The most extreme case is Uruguay, the only country where homework frequency shows a statistically significant negative effect, but one which is compensated for by the opposite effect of homework amount. These data have new educational implications: very large amounts of homework not only seem detrimental (in most countries the effect of homework amount is negative), but there is also evidence indicating that within-class differences between students are greater in those class groups with a larger amount of homework (Fernández-Alonso et al., 2017a).

These results must be interpreted in light of our study’s limitations. The most important of which is probably the lack of a measure of prior performance. In the data, the only variable related to school history was the repetition of a school year, which as one might expect, had a negative effect in every case. However, research has repeatedly shown that measures of previous performance are the best predictors in this type of study (Murillo and Martínez-Garrido, 2013; Núñez et al., 2014; Fernández-Alonso et al., 2015). In addition, the statistical models used in this study are correlational and therefore the conclusions cannot be read in causal terms. As Trautwein and Lüdtke (2009) clearly indicated, the word “effect” must be understood as “predictive effect,” as it is not possible to establish the direction of the association. Our study predicts achievement in science with generic homework measures. It would have been better to employ measures which were specific to the subject being studied (e.g., time spent on science homework, Trautwein and Lüdtke, 2007, 2009). Nevertheless, studies which have looked at the relationship between results in various subjects and specific homework time measures have found similar effect sizes in the subjects they evaluated (Lubbers et al., 2010; Chang et al., 2014). An additional limitation is that although TERCE evaluated two age cohorts: 3rd and 6th year of compulsory education, in the context questionnaire for the 3rd year there was insufficient information to construct variables such as homework time and homework amount. For that reason in this study we only focus on the 6th year sample, something which should be borne in mind when considering the generalizability of the results. Future research must be directed toward including other variables which have been shown to be important. The specification and confirmation of a comprehensive model which addresses student behavior and motivation, homework characteristics, teachers’ use of homework, teaching quality, teacher review and feedback, and the role of the family in homework is an unresolved issue in the Latin American context, while there is already evidence of this type available in other regions (Epstein and Pinkow, 1988; Cooper, 1989; Trautwein et al., 2006; Cunha et al., 2018; León et al., 2018).

## Ethics Statement

This study was carried out in accordance with the recommendations of the United Nations Educational, Scientific and Cultural Organization (UNESCO). All subjects gave written informed consent in accordance with the Declaration of Helsinki.

## Author Contributions

RF-A and JM designed the research. RF-A, PW, and AG-L analyzed the data. MÁ-D and MC interpreted the data. RF-A, PW, AG-L, MÁ-D, and MC drafted the paper. JM revised it critically. All authors gave final approval of the version to be published and have ensured the accuracy and integrity of the work.

## Conflict of Interest Statement

The authors declare that the research was conducted in the absence of any commercial or financial relationships that could be construed as a potential conflict of interest.
